# Using Akaike's information theoretic criterion in mixed-effects modeling of pharmacokinetic data: a simulation study

**DOI:** 10.12688/f1000research.2-71.v2

**Published:** 2014-05-28

**Authors:** Erik Olofsen, Albert Dahan

**Affiliations:** 1 Department of Anesthesiology, Leiden University Medical Center, PO Box 9600, 2300 RC Leiden, Netherlands

**Keywords:** population model, pharmacokinetics, Akaike, information theoretic criterion

## Abstract

Akaike's information theoretic criterion for model discrimination (AIC) is often stated to "overfit", i.e., it selects models with a higher dimension than the dimension of the model that generated the data. However, with experimental pharmacokinetic data it may not be possible to identify the correct model, because of the complexity of the processes governing drug disposition. Instead of trying to find the correct model, a more useful objective might be to minimize the prediction error of drug concentrations in subjects with unknown disposition characteristics. In that case, the AIC might be the selection criterion of choice.

We performed Monte Carlo simulations using a model of pharmacokinetic data (a power function of time) with the property that fits with common multi-exponential models can never be perfect - thus resembling the situation with real data. Prespecified models were fitted to simulated data sets, and AIC and AIC *_c_* (the criterion with a correction for small sample sizes) values were calculated and averaged. The average predictive performances of the models, quantified using simulated validation sets, were compared to the means of the AICs. The data for fits and validation consisted of 11 concentration measurements each obtained in 5 individuals, with three degrees of interindividual variability in the pharmacokinetic volume of distribution.

Mean AIC *_c_* corresponded very well, and better than mean AIC, with mean predictive performance. With increasing interindividual variability, there was a trend towards larger optimal models, but with respect to both lowest AIC *_c_* and best predictive performance. Furthermore, it was observed that the mean square prediction error itself became less suitable as a validation criterion, and that a predictive performance measure should incorporate interindividual variability.

This simulation study showed that, at least in a relatively simple mixed effects modelling context with a set of prespecified models, minimal mean AIC *_c_* corresponded to best predictive performance even in the presence of relatively large interindividual variability.

## Introduction

We first define population data as a set of one or more measurements in two or more individuals ( *e.g.,* patients, volunteers, or animals). Such data may be characterized by mixed-effects models, where the mixed effects consist of fixed and random effects. Fixed effects are, for example, the times at which the measurements are obtained, and covariates such as demographic characteristics of the individuals. When mixed-effects models are fitted to population data, the question arises as to how many of those effects should be incorporated in the model. This is the so-called problem of variable selection ^1^.

One strategy is to observe the change in goodness-of-fit by adding one more parameter and testing the significance of that change ^2^. In the maximum likelihood approach, the objective function value (OFV), being the minus two logarithm of the likelihood function, is minimized. To attain a *p*-value of *e.g.,* 0.05 or less, the decrease in OFV, when adding one parameter, should be 3.84 or more ^2^.

Another strategy is to apply Akaike’s information theoretic criterion (AIC), which can be written as

AIC = OFV + 2⋅D,               (1)

where *D* is the number of parameters in the model ^1– 4^. The model with the lowest value of AIC is considered the best one. In the case of just adding one parameter, the OFV needs to decrease only 2 points or more to be incorporated in the model, so the associated *p*-value > 0.05 seems too high to justify this strategy.

When additional model parameters are incorporated, the significance of one model parameter might change, but the interpretation of AIC does not ^4^. However, when multiple significance tests are performed, the significance level of each individual test should be corrected to a lower value, so a decrease of 2 points for one parameter does again seem to be too low.

Even if the strategy of using AIC leads to optimal variable selection, the question arises as to whether this is also the case when using mixed-effects models. In theory, the model that is best according to AIC is the one that minimizes prediction error ^3, 5^; and this is also true for a mixed effects model when predicting data for individuals for which no data have been obtained so far ^5^.

In the literature, many simulation studies have assessed the performance of AIC, but to our knowledge these were never done in selecting the model with minimal prediction error for population data. In this article, we will define a toy pharmacokinetic model and observe the performance of AIC when adding fixed effects to this model, as well as when adding interindividual variability.

## Methods

### A hypothetical pharmacokinetic model

Consider the following function *y*( *t*), an infinite sum of exponentials, and its relationship with a (negative) power of time ^6^:

$$y(t)=\int_{0}^{\infty }\exp(-\lambda t)d\lambda =-\frac{1}{t}\cdot \exp(-\lambda t){|}_{0}^{\infty }=\frac{1}{t}\;\text{for}\;t>0.\;\;\;\;\;\;\;\;\;\;\text{(2)}$$

**Figure 1.**
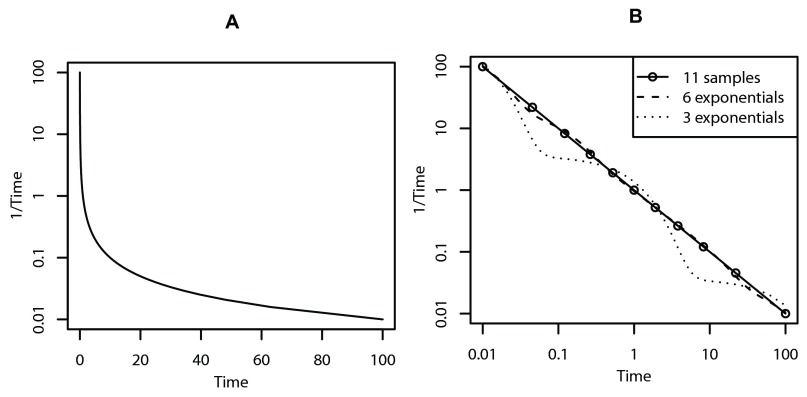
**A**: function *y*( *t*) = 1/ *t*, and **B**: approximations obtained by fitting six and three exponentials to the depicted eleven samples. Note the log-lin and log-log scales for panels **A** and **B**, respectively. Time has arbitrary units.

Figure 1A shows that this function looks like a typical pharmacokinetic profile after bolus administration. This model is to be regarded as a toy model, because we do not expect it to adequately describe pharmacokinetic data, although variations of power functions of time have been shown to fit pharmacokinetic data well ^6^. We approximate *y*( *t*) = 1/ *t* by the following sum of *M* exponentials with *K* nonzero coefficients ***α***:

$$\hat{y}(t_{j};\alpha ,\lambda )=\sum_{m=1}^{M}\alpha_{m}\;\exp(-\lambda_{m}t_{j}).\;\;\;\;\;\;\;\;\;\;\text{(3)}$$

The *M* parameters **λ** are fixed and set as described in the next subsections. This approximation has the property that with while the fit improves with increasing *K*, we would need no less than *K* = *M* exponentials to obtain a perfect fit (with *M* time instants *t _j_*). Moreover, with noisy data, it might be that for *K < M* an optimal fit is obtained in the sense that then the associated prediction error of the model is minimal. Figure 1B shows how eleven (in this case error-free) samples from this function can be approximated by sums of exponentials.

### Individual data modeling and simulation

In the following, the time instants *t _j_*, *j* = 1 *, · · · , M*, centered around 1, were chosen within [1/ *t*_max_ *, t*_max_] according to

$$t_{j}={\left(\frac{j}{M+1-j}\right)}^{\gamma },\;\;\;\;\;\;\;\;\;\text{(4)}$$

with γ = log( *t*_max_)/log( *M*); *t*_max_ was set to 100 (see the time axis of Figure 1B for an example with *M* = 11). Simulated data with constant proportional error were generated *via*

$$y\left(t_{j}\right)=\frac{1}{t_{j}}\left(1+\epsilon_{j}\right),\;\;\;\;\;\;\;\;\;\;\text{(5)}$$

where *ϵ _j_* denotes Gaussian measurement noise with variance *σ* ^2^. The *M* time constants **λ** were fixed according to λ *_m_* = 1/ *t _m_*, *m* = 1 *, · · · , M*. In this setting the model equation (3) can be fitted to simulated data using weighted linear least squares regression, with weight factors *w*( *t _j_*) = 1/ *t _j_* (note that no precaution is needed against *ϵ* ≤ –1). Linear least squares regression is very fast and robust, so it allows for the evaluation of many simulation scenarios.

Simulation scripts: update 1 Scripts used to simulate and analyze the hypothetical pharmacokinetic model data. The aici.R and aicp.R scripts have been updated from http://dx.doi.org/10.6084/m9.figshare.157246. Click here for additional data file. Copyright: © 2014 Olofsen E and Dahan A 2014

### Population data modeling and simulation

Population data consisting of *N* individuals were simulated *via*

$$y_{i}\left(t_{j}\right)=\frac{1}{t_{j}}\cdot \left(\exp\left(\eta_{i}\right)+\epsilon_{ij}\right)\;\text{with}\;i=1,\cdots ,N,\;\;\;\;\;\;\;\;\;\;\text{(6)}$$

where *η _i_* denotes interindividual variability with variance *ω* ^2^. The nonlinear mixed effects model for the population data was then written as:

$${\hat{y}}_{i}\left(t_{j};\alpha ,\lambda \right)=\sum_{m=1}^{M}\alpha_{m}\;\exp\left(-\lambda_{m}t_{j}+\eta_{i}\right).\;\;\;\;\;\;\;\;\;\;\text{(7)}$$

Note that with *N >* 1, a perfect fit is no longer obtained with *K* = *M* nonzero coefficients ***α***, because the *ϵ _ij_* are generally different for different *i* (individuals).

### Statistical analysis

Simulation data were generated *via* equation (6), with random generators in R ^7^. Model fitting was also done in R, with function “lm()” from package “stats”, except for nonlinear mixed-effects model fitting for simulated data with *ω* ^2^ *> * 0, which was done in NONMEM version 7.3.0 ^8^. Parameters ***α*** (see equation (7)) were not constrained to be positive, so that it was not possible for parameters to become essentially fixed to zero, reducing the dimensionality of the model. Prediction error ( *ν* ^2^) was calculated with

$$\nu^{2}=\frac{1}{N\cdot M}{\sum_{i=1}^{N}\sum_{j=1}^{M}\left(\frac{z_{i}\left(t_{j}\right)-{\hat{y}}_{i}\left(t_{j}\right)}{w\left(t_{j}\right)}\right)}^{2},\;\;\;\;\;\;\;\;\;\;\text{(8)}$$

using predictions based on equation (7) with the random effects *η _i_* = 0, and validation data *z _i_*( *t _j_*) also generated *via* equation (6), but with different realizations of *ϵ _ij_* and *η _i_*. Error terms weighted with *w*( *t _j_*) = 1/ *t _j_* are homoscedastic, which is an assumption underlying regression analysis and allows for the interpretation of *ν* ^2^ as independent of time. The objective function OFV was also calculated at the estimated parameters using the validation data, denoted OFV *_v_*, which should on average be approximately equal to Akaike’s criterion (see Supplementary material). OFV *_v_* was compared with AIC and also with Akaike’s criterion with a correction for small sample sizes (AIC *_c_*) ^4^:

$${\text{AIC}}_{C}=\text{OFV}+2\cdot D\cdot (1+\frac{D+1}{N\cdot M-D-1}).\;\;\;\;\;\;\;\;\;\;\text{(9)}$$

The above criteria were normalized by dividing them by the number of observations, and averaged over 1000 runs (unless otherwise stated; and runs where NONMEM’s minimization was not successful were excluded). For plotting purposes, 95% confidence intervals or confidence regions for means were determined using R’s packages “gplots” and “car”, under the assumption that averages over 1000 variables are normally distributed. Model selection frequencies were calculated based on optimal models according to AIC *_c_* as determined for each simulation data set.

### Selection of parameter values

Simulation parameters *M* and *σ* ^2^ are expected to determine the number of exponentials *K*; if the number of measurements *M* increases and/or the measurement error *σ* ^2^ decreases, *K* will increase. Without interindividual variance, so *ω* ^2^ = 0, the information in the data increases as *N* increases, so also in that case *K* is expected to increase. With *N* = 2, *M* = 11 and *σ* ^2^ = 0.5, pilot simulations indicated a *K ≈* 4. When *ω* ^2^ > 0, prediction error will increase, but it is less easy to predict what its effect will be on *K*. For *ω* ^2^ values of 0, 0.1, and 0.5 were selected - values that are encountered in practice. Because there is only one random effect in the mixed effects model, the relatively low number of individuals *N* = 5 was selected.

For a certain choice of *M*, there are 2 *^M^ −* 1 possible combinations of λs to choose for the terms exp(−λ *_m_t _j_*) in the sum of exponentials (excluding the case of a model without exponentials). Because accurate evaluation of all models at different parameter values is not feasible with respect to computer time, the set of possible combinations was reduced to one with evenly spaced λs. Table 1 gives an example for the case *M* = 11.

**Table 1.** Selecting *K* = 1 *, · · · , M* = 11 evenly spaced rate constants from **λ**: 0 and 1 denote *α _m_* to be fixed to zero, and a free parameter to be estimated, respectively (see equation (7)).

K	*m* : 1	2	3	4	5	6	7	8	9	10	11
1	0	0	0	0	0	1	0	0	0	0	0
2	1	0	0	0	0	0	0	0	0	0	1
3	1	0	0	0	0	1	0	0	0	0	1
4	1	0	0	1	0	0	0	1	0	0	1
5	1	0	0	1	0	1	0	1	0	0	1
6	1	0	1	0	1	0	1	0	1	0	1
7	1	0	1	1	0	1	0	1	1	0	1
8	1	1	0	1	1	0	1	1	0	1	1
9	1	1	0	1	1	1	1	1	0	1	1
10	1	1	1	1	1	0	1	1	1	1	1
11	1	1	1	1	1	1	1	1	1	1	1

## Results

**Figure 2.**
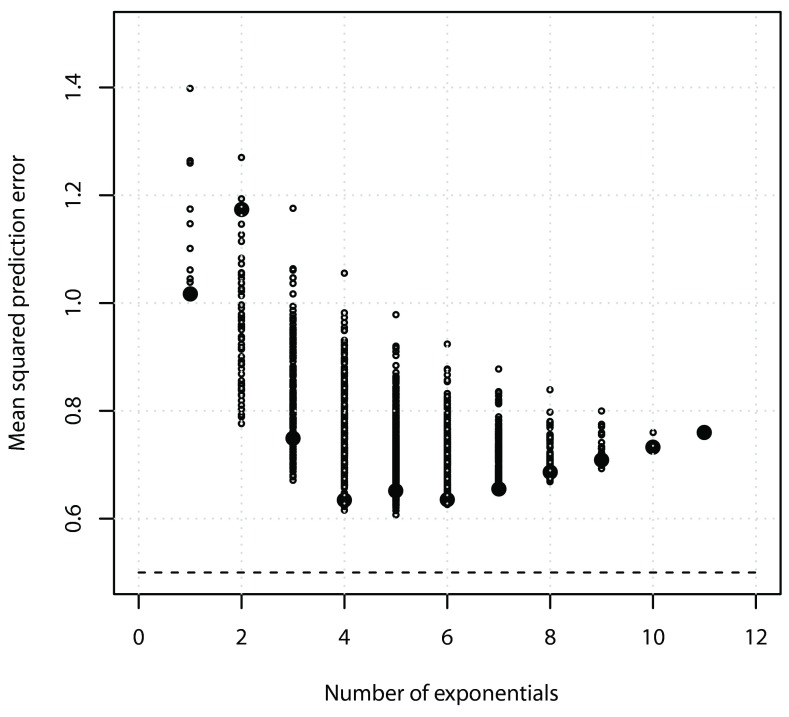
Mean squared prediction error *ν* ^2^ ( equation (8)) as a function of the number of exponentials, with 2047 models, averaged over 100 runs, *N* = 2, *M* = 11, *σ* ^2^ = 0.5, *ω* ^2^ = 0. The dashed line represents the prediction error from the true model, so that *ν* ^2^ = *σ* ^2^. The bigger solid circles correspond to the models chosen in Table 1.

Figure 2 shows the averaged prediction error *versus* number of exponentials for all possible choices of **λ**, with *N* = 2, *M* = 11, *σ* ^2^ = 0.5, and *ω* ^2^ = 0. From the figure it is clear that prediction error may indeed increase if the number of exponentials selected is too large. The bigger solid circles correspond to the models chosen in Table 1; in general the evenly spaced selection of exponents resulted in models with smallest prediction error.

**Figure 3.**
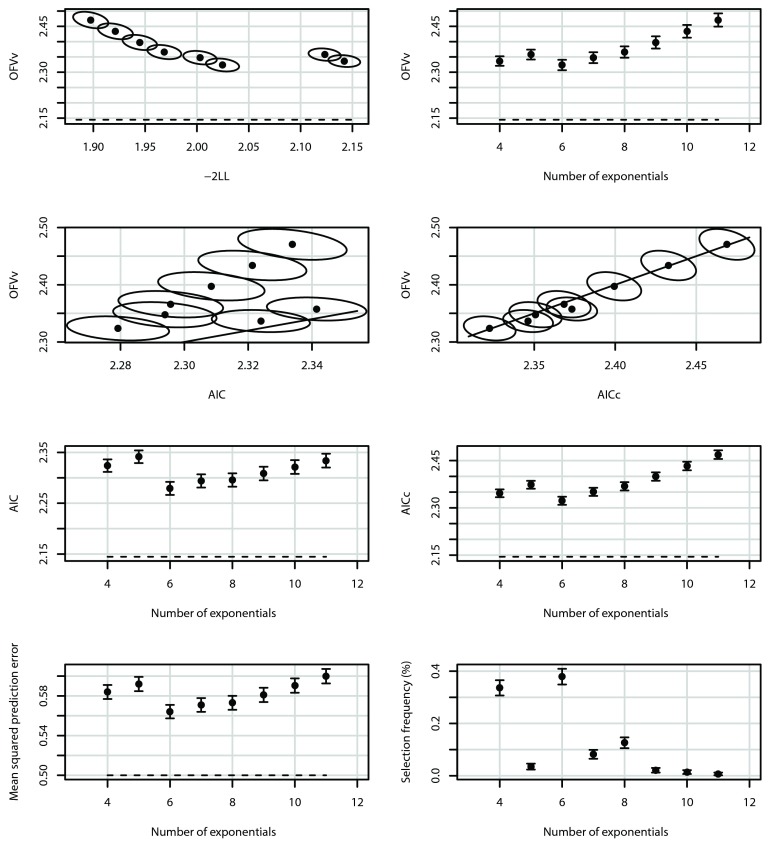
Mean OFV *_v_* as a function minus of two log likelihood (-2LL), the number of exponentials, AIC and AIC *_c_* (top four panels), and AIC, AIC *_c_*, prediction error *ν* ^2^, and model selection frequencies as a function of the number of exponentials (lower four panels), averaged over 1000 runs, *N* = 5, *M* = 11, *σ* ^2^ = 0.5, *ω* ^2^ = 0. The dashed lines represent the theoretical values for an infinite amount of data (see Supplementary material). Error bars and ellipses denote 95% confidence intervals and confidence regions, respectively. The solid lines in the middle upper panels are lines of identity.

Figure 3 shows simulation results using the model set defined in Table 1, starting from *K* = 4, with parameters *N* = 5, *M* = 11, *σ* ^2^ = 0.5, and *ω* ^2^ = 0. The model with *K* = 6 exponentials had minimal mean AIC *_c_*, and also minimal mean OFV *_v_* and minimal prediction error *ν* ^2^. With *N* = 5, *M* = 11, there are still visible differences between AIC *_c_* and AIC; although AIC would in this case also select the optimal model, AIC appears to favor more complex models. Note that the sizes of the confidence intervals and confidence regions can be made arbitrarily small by choosing the number of runs to be higher than the selected number of 1000 (at the expense of computer time).

**Figure 4.**
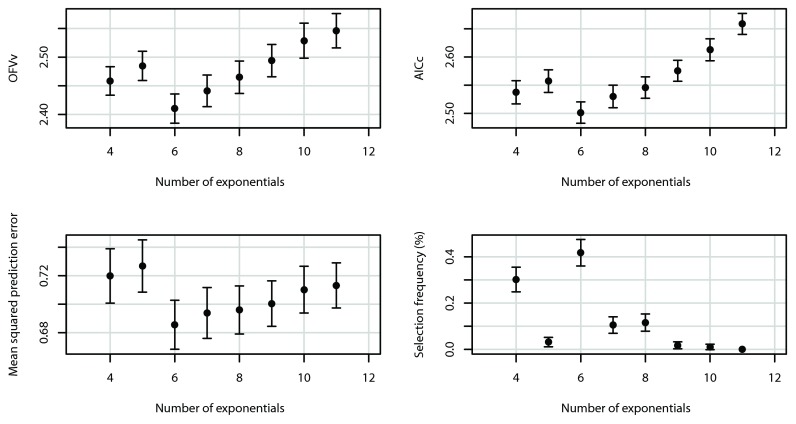
Mean OFV *_v_*, AIC *_c_*, prediction error *ν* ^2^, and model selection frequencies as a function of the number of exponentials, for *ω* ^2^ = 0.1; parameters otherwise identical.

Figure 4 shows simulation results with *ω* ^2^ = 0.1; mixed-effects analysis was used to fit the population data. The main difference with the results of data with *ω* ^2^ = 0 is the overall increase in OFV *_v_* and AIC *_c_*. The optimal number of exponentials remained *K* = 6.

**Figure 5.**
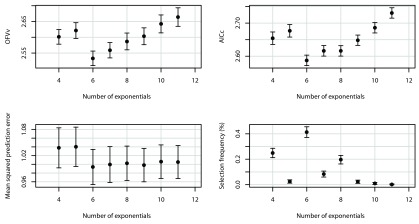
Mean OFV *_v_*, AIC *_c_*, prediction error *ν* ^2^, and model selection frequencies as a function of the number of exponentials, for *ω* ^2^ = 0.5; parameters otherwise identical.

Figure 5 shows simulation results with *ω* ^2^ set at the higher value of 0.5. The main differences with the results of data with *ω* ^2^ = 0.1 are again the overall increase in OFV *_v_*, AIC *_c_* and prediction error, and also in the variability in the prediction error. The optimal number of exponentials remained *K* = 6, although AIC *_c_* begins to favor the models with larger *K* (a simulation with *N* increased to 7, both OFV *_v_* and AIC *_c_* favored larger models; data not shown).

## Discussion

With the objective of creating a simulation context resembling pharmacokinetic analysis where concentration data are approximated by a sum of exponentials, the toy model *y*( *t*) = 1/ *t* was chosen. In this setting, reality - the reality of the toy model - is always underfitted. When mixed effects models were fitted to simulated data, mean AIC *_c_* was approximately equal to the validation criterion mean OFV *_v_*, and their minima coincided. With large interindividual variability, mean expected prediction error ( *ν* ^2^, see equation (8), with random effects fixed to zero), was less discriminative between models, so that it becomes less suitable as a validation criterion; it does not take into account whether estimated interindividual variability matches the variability in the validation data.

### Akaike’s *versus* the conditional Akaike information criterion

Vaida and Blanchard proposed a conditional Akaike information criterion to be used in model selection for the “cluster focus” ^5^. It is important to stress that their definition of cluster focus is the situation where data are to be predicted of a cluster that was also used to build the predictive model. In that case, the random effects have been estimated, and then the question arises how many parameters that required. In our situation, a cluster is the data from an individual; AIC was used in the situation of predicting population data consisting of individual data that were not used to build the model. This would seem to be the most common situation in clinical practice. Furthermore, AIC for the population focus is asymptotically equivalent with leave-one-individual-out cross-validation; AIC for the individual focus with leave-one-observation-out cross-validation ^9^.

### Akaike’s *versus* the Bayesian information criterion

We chose to perform simulations using the model given by equation (2) because approximating data with a sum of exponentials is daily practice in pharmacokinetic analysis where data are obtained from “infinitely complex” systems, and we cannot hope to find the “correct” model. The Bayesian information criterion (BIC) is consistent in the sense that it selects the correct model, given an infinite amount of data ^4^. The reason that AIC can be used in “real-life” problems is that as the amount of data goes to infinity, the complexity, or dimension, of the model that should be applied should also go infinity ^10^. Burnham and Anderson show that it is possible to choose the prior for BIC in such a way that it incorporates the knowledge that more complex models should be favored if the amount of data increases, and so that the BIC “reduces” to AIC ^4, 10^. In the situation that the correct model set belongs to the set of evaluated models, a selection criterion that both finds the correct model and minimizes prediction error would be preferable - but Yang concluded that this may not be possible ^11^. As the correct model is most likely not included in the set chosen by the modeler, using AIC - or optimizing predictive performance - seems preferable. The expression of the BIC contains a factor log( *N'*) instead of 2 with AIC, where *N'* is the effective sample size. *N'* depends on the association between parameters and random effects ^12^, and is for our model definition of the order *N'* = ( *D* − 2) *· N · M* + 2 *· N*. This indicates that if *N'* ≉ 7.4, a model with minimum BIC has worse predictive properties than a model with minimum AIC.

### Model selection criterion AIC and predictive performance

Intuitively, predicting data for an individual that cannot be “individualized” seems problematic; because the data are predicted using a random effect *η _i_* set to zero, instead of the value fitting for that individual. However, AIC is related to the expected model output; and for individual data not used in building the predictive model, the expected model is output is obtained with mixed effects set to zero, although nonlinearities may bias expectation - but this is also true for nonlinear models without mixed effects.

Furthermore, it should be noted that minimizing AIC has a more general interpretation, namely optimally capturing the information contained in the data ^4^. Independent or future population data *z* are not just predicted by $\hat{y}$; also the distributions of the expected random effects *ϵ* and *η* are characterized by ${\hat{\sigma }}^{2}$ and ${\hat{\omega }}^{2}$. That is why OFV *_v_* (and not *ν* ^2^) is the criterion to be used to assess the predictive performance of a model.

In pharmacokinetic analysis, it may not really be the most appropriate test (using a hypothesis test assuming a *X* ^2^ distribution for the objective function) of whether an added exponential is statistically significant ^13^. Here the hypothesis *H*_0_: the data originate from a *K*-exponential model (and *H*_A_: the data originate from a higher dimensional model) is almost certain to be false. Furthermore, when taking a low *p*-value, it is also almost certain that the model selected has worse predictive properties. If a model is to be applied in clinical practice, for example for drug administration in a patient never studied before, the model should be as predictive as possible. However, it may be sensible to test whether a certain fixed effect has both a clinically and statistically significant effect, if it is costly to reach a false conclusion, for example in case of increased risks for patients, or in the field of drug development.

### Regression weights as functions of the model output

The simulated data were analyzed using weighted (non)linear regression, see equation (6), where measurement noise was weighted according to the exact function value. In practice, when the weights are unknown, a choice must be made to weight the data according to the measurements or to the model output, depending on which is likely to be the most accurate. To match the latter case, simulated data should be generated ( *cf.* equation (6)) *via*

$$y_{i}\left(t_{j}\right)=\frac{1}{t_{j}}\cdot \exp\left(\eta_{i}\right)\cdot \left(1+\epsilon_{ij}\right).\;\;\;\;\;\;\;\;\;\;\text{(10)}$$

The likelihood function and AIC are both still well-defined if the model output $\hat{y}$ *_i_*( *t _j_*) ≠ 0. Prediction errors are to be calculated with

$$v^{2}=\frac{1}{N\cdot M}\sum_{i=1}^{N}{\sum_{j=1}^{M}\left(\frac{z_{i}\left(t_{j}\right)-{\hat{y}}_{i}\left(t_{j}\right)}{{\hat{y}}_{i}\left(t_{j}\right)}\right)}^{2},\;\;\;\;\;\;\;\;\;\;\text{(11)}$$

where $\hat{y}$ possibly becomes arbitrarily close to zero for less than optimal models, and *ν* ^2^ may be based on long-tailed distributed numbers. To be able to compare prediction errors from different models, the weight factors could be chosen identical for all *K* to the model output of the largest model - see the Supplementary material for further analysis.

### Model selection uncertainty

Theoretically, and in the discussed simulations, minimum mean AIC is related to best mean predictive performance, where the mean is taken across multiple studies and prespecified models. This holds independent of the number of models. However, in practice, we have data from one study and the task of specifying the models to consider. As soon as there is more than one model, there is a nonzero probability that the model selected based on AIC would have, on average, a larger prediction error than the optimal one. Also if we were able to repeat the study, the average prediction error based on the models with minimum AIC would be larger than optimal. With many models, model selection is called unstable in the sense that each time a study is repeated it would lead to the selection of another model.

The figure panels with the model selection frequencies (in Figure 3, Figure 4, and Figure 5) show: 1) there is relationship between the model with highest selection probability and minimum mean prediction error, but this relationship is not one-to-one; 2) there can be an almost as large selection probability for a model that is not associated with minimum mean prediction error; but 3) in that case, their minimum mean prediction errors are comparable.

Models with equal mean predictive properties may have different properties in different extrapolation scenarios. Model averaging ^4^, where model parameters or their predictions are averaged, reduces model selection instability and hence may be used to avoid model specific inference which discards model selection uncertainty. Data dredging ^4^ refers to the situation where there is an increasingly large set of models which are not prespecified. At the point the data dredging is stopped (by the investigator, or by the computer), the best model is at high risk to fit only the data at hand, and hence cannot be used for prediction ^14^.

### Limitations of the study

We recognize the following limitations of our study:

- The simulation model contained only one random effect to describe interindividual variability, and therefore the number of random effect (co)variances was fixed to one in the model set used for fitting. While the number of (co)variance parameters should be counted as ordinary parameters ^5^, at least in well behaved situations ^15^, we did not investigate the process of optimizing this part of a random effects model.
- The nonlinearity in the mixed-effects model was simply due to a multiplicative factor exp( *η*) in the model output. Usually, random effects in pharmacokinetic models have more complex influence on the model output. However, the lognormal nature of exp( *η*) is a characteristic property of both our toy model and general pharmacokinetic models.
- The characteristics of the exponentials incorporated in the regression models were evenly spaced, and the values of the rate constants **λ** were fixed. We expect that with more freedom in the specification of the set of models, prediction errors with overfitted models may be worse. However, the agreement between AIC *_c_* and prediction error should persist.
- We did not evaluate all possible models within their definition, but only those listed in Table 1, and it makes sense to limit the model set to reduce model selection instability ^4, 11^. We did not address how to optimally select the rate constants **λ**. Stepwise selection methods have their disadvantages ^13^. With stepwise forward selection, AIC *_c_* may even perform worse than AIC ^16^.
- We did not evaluate the process of covariate selection. However, the set of exponentials may be viewed as a number of (somewhat correlated) predictors. It is therefore expected that the present findings also hold for other types of covariates.

## Conclusion

In conclusion, the present simulation study demonstrated that, at least in a relatively simple mixed effects modeling context with a set of prespecified models, minimum mean AIC *_c_* coincided with best predictive performance, also in the presence of interindividual variability.

## Software availability

figshare: Simulation scripts: update 1, doi: 10.6084/m9.figshare.1036483 ^17^

## Supplementary material

In the following, we summarize theory on the maximum likelihood approach and AIC relevant for this paper. We start with the situation for data from one individual and show how AIC is related to OFV *_v_*. Subsequently we discuss the situation for population data.

Suppose the model for measured data *y _j_*, *j* = 1 *, · · · , M* is given by ( *cf.* equation (5), equation (6), and equation (10))

$$y_{i}={\hat{y}}_{j}+w_{j}.\epsilon_{j},\;\;\;\;\;\;\;\;\;\;\text{(12)}$$

where $\hat{y}$ *_j_* is the model output, *w _j_* are weight factors, and *ϵ _j_* are independent normally distributed with mean zero and variance *σ* ^2^. The likelihood function *L* for this data set is then given by

$$L(y;\theta )=\prod_{j=1}^{M}\frac{1}{w_{j}\sigma \sqrt{\left(2\pi \right)}}\exp\left[-\frac{1}{2}{\left(\frac{y_{j}-{\hat{y}}_{j}}{w_{j}\sigma }\right)}^{2}\right],\;\;\;\;\;\;\;\;\;\;\text{(13)}$$

where the set of parameters ***θ*** contains *σ* ^2^ and those needed to calculate $\hat{y}$. The objective function value (OFV) is defined as minus two times the natural logarithm of the likelihood:

$$\text{OFV}=-2\log(L(y;\theta ))=\sum_{j=1}^{M}\log\left(w_{j}^{2}\right)+M\log\left(\sigma^{2}\right)+M\log\left(2\pi \right)+\frac{1}{\sigma^{2}}{\sum_{j=1}^{M}\left(\frac{y_{j}-{\hat{y}}_{j}}{w_{j}}\right)}^{2}.\;\;\;\;\;\;\;\;\;\;\text{(14)}$$

Note that in writing “OFV”, the data and parameters it depends on have been omitted. Now maximum likelihood is obtained when OFV is minimal; constant terms such as *M* log(2 *π*) may then be discarded (for example, in NONMEM’s calculation of the the objective function). The minimum is attained for certain values of parameters of $\hat{y}$, and for the parameter value of *σ* ^2^, when the derivative of *OFV* with respect to that parameter is zero:

$$\frac{\partial \text{OFV}}{\partial \sigma^{2}}=\frac{M}{\sigma^{2}}-\frac{1}{{\left(\sigma^{2}\right)}^{2}}{\sum_{j=1}^{M}\left(\frac{y_{j}-{\hat{y}}_{j}}{w_{j}}\right)}^{2}=0,\;\;\;\;\;\;\;\;\;\;\text{(15)}$$

so the maximum likelihood estimator of *σ* ^2^ is

$${\hat{\sigma }}^{2}=\frac{1}{M}{\sum_{j=1}^{M}\left(\frac{y_{j}-{\hat{y}}_{j}}{w_{j}}\right)}^{2}\;.\;\;\;\;\;\;\;\;\;\;\text{(16)}$$

By subsituting this estimate in equation (14), we obtain

$$\text{OFV}=\sum_{j=1}^{M}\log\left(w_{j}^{2}\right)+M\log\left({\hat{\sigma }}^{2}\right)+M\log\left(2\pi \right)\;+\;M.\;\;\;\;\;\;\;\;\;\;\text{(17)}$$

By substituting this result in equation (1), we have

$$\text{AIC}=\sum_{j=1}^{M}\log\left(w_{j}^{2}\right)+M\log\left({\hat{\sigma }}^{2}\right)+M\log\left(2\pi \right)+M+2D.\;\;\;\;\;\;\;\;\;\;\text{(18)}$$

The term 2 *D* arises from the fact that in minimizing the Kullback-Leibler information, *i.e.,* a measure of the distance between reality and the best approximating model, expectations have to be taken over a data space leading to estimates of parameters ***θ*** (and hence $\hat{y}$, and possibly *w* (see below)) and over a second independent data space *y* ^4^. So AIC as defined above should on average be approximately equal the value of OFV ( equation 14), with estimated values for the parameters and validation data *z _j_*, denoted OFV *_v_*:

$${\text{OFV}}_{V}=\sum_{j=1}^{M}\log\left(w_{j}^{2}\right)+M\log\left({\hat{\sigma }}^{2}\right)+M\log\left(2\pi \right)+\frac{1}{{\hat{\sigma }}^{2}}.{\sum_{j=1}^{M}\left(\frac{z_{j}-{\hat{y}}_{j}}{w_{j}}\right)}^{2}.\;\;\;\;\;\;\;\text{(19)}$$

So when OFV and AIC are both minimized, the latter term - the sum of squared weighted prediction errors - should also be minimal. For the plots in this paper, the measures OFV, OFV *_v_*, AIC, and AIC *_c_*, were normalized by dividing them by the number of data samples. With an infinite amount of data, and ${\hat{\sigma }}^{2}$ = *σ* ^2^, the normalized criteria should attain the value of log( *σ* ^2^)+log(2 *π*)+1.

Note that if the weights *w _j_* are taken as in subsection “Data simulation”, the term Σlog( *w _j_* ^2^) vanishes (this is a just a curiosity of that choice of weights); if the *w _j_* are taken as the measurements *y _j_*, the expectation of this term is the same for every *K* (for every model considered here). However, if the weights are taken as the model output $\hat{y}$ *_j_*, the expectation of the term will not vanish for a less than perfect model, and will differ between different models. To compare their *ν* ^2^, the weights for all models could be fixed to the model output of the best model - but since that is unknown at this point - to the output of the largest model.

For population data, the likelihood function is the product across individual marginal likelihoods where the random effects *η* contained in equation (13), when $\hat{y}$ is given by equation (6), have been integrated out. Usually, these integrals need to be numerically approximated, *e.g.*, by NONMEM. So the context of AIC is then also the one where the *η*s have been integrated out (but with the parameters at their estimated values), which is to be done when all data are acquired. So while the characteristics of the set of (validation) data are optimally captured, this context is different from the case where prediction errors are calculated with the random effects set to zero instead of integrated out. In that case, the above AIC and OFV *_v_* criteria do not match, as the components of the likelihood in equation (13) are no longer independent (they can only independent if the true values of *η* for the individuals are also zero). Note however, that from the higher perspective of optimally characterizing a future set of population data, this is a less important case.

Finally, it should be noted that the parameter estimates may not be consistent ( *i.e.,* do not converge to their true values when the amount of data goes to infinity if the $\hat{y}$ *_j_* do not properly account for heteroscedasticity ^18^. In the derivation of AIC ^4^, it is only required that the likelihood function is maximized; consistency is not required.
