# Supplementary material for: Using Akaike's information theoretic criterion in mixed-effects modeling of pharmacokinetic data: a simulation study
Source: F1000Res. 2014 May 28;2:71. Originally published 2013 Mar 4. [Version 2] doi: 10.12688/f1000research.2-71.v2 (PMC4670010; doi:10.12688/f1000research.2-71.v2)
Supplement: Peer review history

# Peer review: Referee response for version 2

**DOI:** 10.5256/f1000research.4469.r4893

**Published:** 2014-08-22

**Authors:** Paul Eilers

**Affiliations:** 1 Department of Biostatistics, Erasmus Medical Center, Rotterdam, Netherlands

The explanation in the authors' response, about the effective degrees of freedom, clarifies the matter. That will be helpful to the readers.

I noticed a typo in the second paragraph of the Introduction, explaining the OFV. Change to "... (OFV), being minus two times the logarithm ...".

I have read this submission. I believe that I have an appropriate level of expertise to confirm that it is of an acceptable scientific standard.

# Peer review: Referee response for version 2

**DOI:** 10.5256/f1000research.4469.r5213

**Published:** 2014-06-24

**Authors:** Julie Bertrand

**Affiliations:** 1 UCL Genetics Institute, University College London, London, UK

**Abstract:**

- *"[W]ith three degrees of inter... volume of distribution"* This does not make sense, as no mention of the volume parameter is made afterward in the body of the text.

 

**Introduction:**

- *"Fixed effects are ... the measurements obtained"*, Usually the fixed effects are the population/average/mean values of the model parameters, so this sounds incorrect perhaps because it's out of context.

 

**Methods:**

- The authors should be clearer about the model parameters that are estimated : K non-zero parameters α _m_ (?),as K doesn't even appear in equation (3)?
- What are those α _m_? Do they correspond to dose/volume in an IV bolus equation? Then I'm not sure one can identify M in such parameters?
- " *Note that with N > 1, a perfect fit is no longer obtained with K = M nonzero coefficients α, because the ϵij are generally different for different i (individuals)"* The authors should add that it is the case here because in their model there is only one random effect per individual.
- The constraint/absence of constraint on the α _m_ is not clear; those parameters were not constrained so they could be equal to zero? The authors should be more explicit.
- *"[U]sing predictions based on equation (7) with the random effectsηi= 0, and validation data zi(tj) also generated via equation (6), but with different realizations of ϵij and ηi" *It is not clear to me how are those predictions were obtained? How are the random effect and residual errors generated (type and parameter values of the distributions)?
- *"The above criteria were normalized by dividing them by the number of observations, and averaged over 1000 runs"* Seems incorrect or not explained enough. In mixed effect models you cannot compare two designs soley on the total number of observations. The information is not the same for n _tot_=20 resulting from i) 2 subjects with 10 samples each and ii) 5 subjects with 4 samples each.
- In Figure 3, the plots of OFV versus -2LL, AIC and AICc do not make sense to me as the relationship is evident from the equations, plus there is no explanation about what the separate points correspond to. 

**Discussion:**

- I am not convinced by the section on BIC as the claims are rather strong in absence of comparison on the simulations. I suggest the authors shorten it.
- The first section of "Model selection criterion AIC and predictive performance" does not make sense to me, are the authors talking about AIC capability to select a model that would predict new subjects? I thought that was the criteria they actually used.
- Also in "Model selection criterion AIC and predictive performance" the last section seems full of strong claims again (as for BIC) not justified through the present simulation study; I would suggest the authors remove the 1st and last sections I've mentioned.
- *"In practice, when the weights are unknown, a choice must be made to weight the data according to the measurements or to the model output, depending on which is likely to be the most accurate.*" There is literature about NOT using the measurements but the model output, that should be corrected.

I have read this submission. I believe that I have an appropriate level of expertise to confirm that it is of an acceptable scientific standard, however I have significant reservations, as outlined above.

# Response

**Published:** 2014-06-30

**Authors:** Erik Olofsen

**Affiliations:** Department of Anesthesiology, Leiden University Medical Center, Netherlands

We thank the reviewer for pointing out those parts of the text that are unclear. Changes to the article will be based on the following observations.

- The simulated interindividual variation influences the concentration level, which is related to volume, and no other disposition characteristics - an explanation will be added to the description of the population model.
- The term "fixed effect" is perhaps not well defined in the literature. In the linear fixed effects model Y = X.β, X (the design matrix) as well as β (the coefficients) are sometimes called fixed effects. In NONMEM's guide V, time - which would be part of the design matrix - is called a fixed effect. Time influences the model output in a non-random manner; how it influences the output depends on the model, and on model parameters. Therefore, a population value of a model parameter is similar to the value of a "fixed effect parameter", and then time could be called a "fixed effect factor".
- The coefficients α are related to disposition characteristics and are parameters to be estimated. This was stated explicitly only in the legend of Table 1, so we agree this should be better explained. Because of the link between the considered sum of exponentials and the integral in equation (2), one would expect the α to be positive, but this constraint was not imposed. The M time constants λ are fixed and have distinct values, so that M coefficients α are identifiable. M-K coefficients were fixed to zero according to Table 1, to obtain models with K free parameters. Indeed, to obtain a perfect fit with more than one individual, M random effects are needed.
- The log likelihood function with known parameter values and homoscedastic random effects is both linear in the number of observations per subject and in the number of subjects. Then the expected values of the log likelihoods for the two designs that you give are equal. The ratio of the estimated log likelihood and the total number of observations is a measure of the entropy in the data due to the random effects (the dashed lines in figure 3), with a deviation due to estimation error (possibly different for the two designs). But although this is interesting, the normalization is indeed also confusing and not essential for our study.
- The plot of OFVv versus -2LL should demonstrate that a lower objective function does not imply a lower prediction error and the plots of OFVv versus AIC and AICc that it is AICc, and not AIC, which corresponds closely to prediction error. Only plots of AIC and AICc versus -2LL would be relatively trivial. We agree that the number of exponentials should be indicated next to the dots.
- We expect the claims on BIC versus AIC in the literature to hold for mixed effect models, but we we agree that the range of effective sample sizes used in the present study is not sufficient to provide firm additional support.
- The first section of "Model selection criterion AIC and predictive performance" is best deleted and the second section slightly rewritten. Interindividual variation in a new set of data is predicted by a distribution rather than only its mode.
- To compare models with AIC in weighted regression, the weights should be the same, which is true when using the measurements, and not so when using the model output as weights, because the models are different. Therefore the model output of the best model could be used as weights. On the other hand, the output of the models should be quite similar, so that the postulated likelihood function approximately holds for the data, which might not hold as well when using the measurements as weights.

# Response

**Published:** 2015-03-18

**Authors:** Erik Olofsen

**Affiliations:** Department of Anesthesiology, Leiden University Medical Center, Netherlands

The normalization of the likelihood by dividing by the total number of measurements was done to have a "target" value for the estimates indicated in the figures. When the between individuals variance is zero, the normalized value is independent of the number of measurements and individuals. When the between individuals variance is greater than zero, and the number of measurements per individual *M* is finite, the normalized value is larger, and in a nonlinear fashion depending on *M*, than this target. Therefore the two designs mentioned above are indeed different. This will be addressed in the next version.

# Peer review: Referee response for version 1

**DOI:** 10.5256/f1000research.869.r1693

**Published:** 2013-10-21

**Authors:** Julie Bertrand

**Affiliations:** 1 UCL Genetics Institute, University College London, London, UK

The title should indeed specify that this work focuses on pharmacokinetics (PK). However I must add that the model function considered is unusual enough that it seems difficult to extend their conclusions to a real PK study analysis.

The abstract is too general and more details should be provided on the simulation study (model function, number of samples, number of subjects, number of random effects) and the results (differences between selection on OFV, AIC and AICc, impact of increasing the random effect variance).

The whole methodology is very well described. But one aspect is missing, as underlined by the other reviewer: the (very direct here) link with the best sum of exponential model and the information in the design. I was not much surprised that K=6 (or 5) exponential got the best AIC when you have 11 evenly spaced samples and the candidate models all had evenly spaced rate constants. Also, why not investigate the performance of BIC (with log(N) and log(NxM)) ?

Finally, the conclusions are balanced in the sense that the authors have rightly identified the limit of their exercise which is the generalization of their results to a real PK data analysis: only one random effect, no covariance parameters, only slope parameters, etc...

I have read this submission. I believe that I have an appropriate level of expertise to confirm that it is of an acceptable scientific standard, however I have significant reservations, as outlined above.

# Peer review: Referee response for version 1

**DOI:** 10.5256/f1000research.869.r928

**Published:** 2013-05-07

**Authors:** Frank Harrell

**Affiliations:** 1 Department of Biostatistics, Vanderbilt University School of Medicine, Nashville, TN, USA

The title and abstract do not place the potentially useful study in the right context. 'Population' can have many meanings and the scope is too wide. Consider narrowing the implied scope to mixed effects PK modeling.AIC can be an excellent metric for selecting from among a very limited number of models. If used in a stepwise process it can result in all the severe problems that stepwise variable selection has. The authors need to be much more careful about multiplicity and model uncertainty. This needs to be carefully discussed, and the authors would add to the literature if they can derive the maximum number of models that can be compared with AIC before the method breaks down.

I have read this submission. I believe that I have an appropriate level of expertise to state that I do not consider it to be of an acceptable scientific standard, for reasons outlined above.

# Peer review: Referee response for version 1

**DOI:** 10.5256/f1000research.869.r809

**Published:** 2013-03-05

**Authors:** Paul Eilers

**Affiliations:** 1 Department of Biostatistics, Erasmus Medical Center, Rotterdam, Netherlands

This is an interesting and useful study, written in a relaxed style. I believe we need more studies like this, because there is a lot of folklore around AIC, mainly claiming that is generally over-fits.

I have one major objection: the effective dimension (ED) of a mixed model is less than the number of parameters (D), because shrinking takes place. The easiest way to obtain ED is to compute the trace of the “hat” matrix. In linear models this is relatively easy. In non-linear models it is harder. What one needs is (in LaTeX notation) $h_{ii} = \partial \hat y_i/ \partial y_i$. I can provide more details if needed. I don’t know if NONMEM can provide the needed quantities.

I have read this submission. I believe that I have an appropriate level of expertise to confirm that it is of an acceptable scientific standard, however I have significant reservations, as outlined above.

# Response

**Published:** 2013-03-05

**Authors:** Erik Olofsen

**Affiliations:** Department of Anesthesiology, Leiden University Medical Center, Netherlands

Thank you for your comments. Vaida and Blanchard (ref. 5 above) discuss two settings: model focus and cluster focus. In the former setting, the effective number of parameters equals the number of fixed effects parameters and variance components (p.354); in the latter setting, the effective number of parameters needs to be estimated in the way you outlined. The first setting, corresponding to the situation of predicting data of "new" subjects, is the one for which the study results should be valid.
